# Counts of hyaluronic acid-containing extracellular vesicles decrease in naturally occurring equine osteoarthritis

**DOI:** 10.1038/s41598-022-21398-8

**Published:** 2022-10-20

**Authors:** Anne-Mari Mustonen, Nina Lehmonen, Sanna Oikari, Janne Capra, Marja Raekallio, Anna Mykkänen, Tommi Paakkonen, Kirsi Rilla, Tytti Niemelä, Petteri Nieminen

**Affiliations:** 1 Faculty of Health Sciences, School of Medicine, Institute of Biomedicine, University of Eastern Finland, P.O. Box 1627, 70211 Kuopio, Finland; 2 Department of Environmental and Biological Sciences, Faculty of Science and Forestry, University of Eastern Finland, P.O. Box 111, 80101 Joensuu, Finland; 3 Department of Equine and Small Animal Medicine, Faculty of Veterinary Medicine, University of Helsinki, P.O. Box 57, 00014 Helsinki, Finland; 4 Cell and Tissue Imaging Unit, Faculty of Health Sciences, School of Medicine, Institute of Biomedicine, University of Eastern Finland, P.O. Box 1627, 70211 Kuopio, Finland

**Keywords:** Biomarkers, Diseases, Medical research, Pathogenesis

## Abstract

Osteoarthritis (OA) is a degenerative joint disease with inadequately understood pathogenesis leading to pain and functional limitations. Extracellular vesicles (EVs) released by synovial joint cells can induce both pro- and anti-OA effects. Hyaluronic acid (HA) lubricates the surfaces of articular cartilage and is one of the bioactive molecules transported by EVs. In humans, altered EV counts and composition can be observed in OA synovial fluid (SF), while EV research is in early stages in the horse—a well-recognized OA model. The aim was to characterize SF EVs and their HA cargo in 19 horses. SF was collected after euthanasia from control, OA, and contralateral metacarpophalangeal joints. The SF HA concentrations and size distribution were determined with a sandwich-type enzyme-linked sorbent assay and size-exclusion chromatography. Ultracentrifugation followed by nanoparticle tracking analysis (NTA) were utilized to quantify small EVs, while confocal laser scanning microscopy (CLSM) and image analysis characterized larger EVs. The number and size distribution of small EVs measured by NTA were unaffected by OA, but these results may be limited by the lack of hyaluronidase pre-treatment of the samples. When visualized by CLSM, the number and proportion of larger HA-containing EVs (HA–EVs) decreased in OA SF (generalized linear model, count: *p* = 0.024, %: *p* = 0.028). There was an inverse association between the OA grade and total EV count, HA–EV count, and HA–EV % (r_s_ = – 0.264 to – 0.327, *p* = 0.012–0.045). The total HA concentrations were also lower in OA (generalized linear model, *p* = 0.002). To conclude, the present study discovered a potential SF biomarker (HA–EVs) for naturally occurring equine OA. The roles of HA–EVs in the pathogenesis of OA and their potential as a joint disease biomarker and therapeutic target warrant future studies.

## Introduction

Lameness is a major factor leading to poor performance and early retirement of equine athletes, and osteoarthritis (OA) is among its most common causes^1^. Similar to the human condition, equine OA is a multifactorial degenerative disease affecting the whole joint^2^. It is characterized by the destruction of articular cartilage and alterations in the subchondral bone and soft tissues of the joint, leading to pain and functional limitations^1^. Joints affected by OA include, for instance, carpal, metacarpophalangeal, proximal/distal interphalangeal, carpometacarpal, tarsal, and femorotibial joints^3^. Prior trauma to the joint, immobilization, poor conformation, improper shoeing, and aging can contribute to the development of OA^1^, which may occur early in the life of equine athletes or later in horses having less intensive exercise^2^. Molecules that are associated with OA and/or experimentally-induced synovitis in horses include cytokines, other inflammatory mediators (*e.g.*, chemokines, prostaglandin E_2_, nitric oxide), and cartilage-degrading proteinases^1,4^.

The currently available medical treatment for OA mostly offers symptomatic relief but fails to repair cartilage damage that is already present^5^. For this reason, early diagnosis before substantial joint pathology would be pivotal, and specific and sensitive biomarkers are actively sought for early-stage OA. Similar to humans, equine OA can occur spontaneously or post-traumatically^6^. Among existing animal models, equine (stifle) articular cartilage shows much promise, as its average thickness, cellular structure, as well as biochemical makeup and properties exhibit the most similarity to human cartilage. The possibility of repeated collection of substantial amounts of synovial fluid (SF) also makes the horse an attractive model for translational OA research in addition to veterinary interest. Biomarkers for equine OA are actively screened^7^ and, regarding SF, pro-inflammatory cytokines, chemokines, cartilage degeneration markers^4,8^, proteomics^9^, eicosanoids^10^, extracellular vesicles (EVs), EV phospholipids^11^, and EV small non-coding RNAs^12^ have emerged as candidates for OA or synovitis, but reliable and easily accessible analyses to detect and monitor equine OA are still lacking.

EVs are nano- and micro-sized membrane-bound particles released by virtually all cell types in physiological and pathological conditions^5^. They constitute a heterogenous mixture of subpopulations with different size and biogenesis (exosomes EXOs, microvesicles MVs, apoptotic bodies ABs, etc*.*). Here, the concept of EV is used as the collective term for all classes of cell-derived vesicles present in equine SF. EVs act as carriers of bioactive molecules and they can modulate the gene expression patterns and behaviour of target cells. Fibroblast-like synoviocytes (FLSs) play a central role in inflammation and cartilage degradation in joint diseases, and EVs have recently emerged as potential conveyors of these effects. SF EVs from arthritic patients can induce pro-inflammatory and catabolic effects on FLSs and chondrocytes but EVs, especially those from mesenchymal stem cells (MSCs), can also provide with anti-OA influence. FLSs are known to secrete EVs that transport hyaluronan (HA) cargo (HA–EVs)^13^. HA is a major glycosaminoglycan constituent of SF produced by FLSs^14^. It provides boundary lubrication, and its SF concentration and molecular size decrease in arthritic patients^15^.

The roles of HA–EVs have not been in the focus of OA research on horses, while SF EV counts increased in experimentally-induced synovitis, and especially the levels of cluster of differentiation 44 (CD44)-positive EVs were elevated in horse SF^11^. Regarding EV lipidomics, short-chain carboxylic acid *N*-modified phosphatidylserine molecules may be involved in synovitis. When chondrocytes were incubated with SF EVs, there was downregulation of genes involved in joint inflammation^11^, and by using EVs from equine bone marrow MSCs, the expression of cartilage-degrading proteinases could decrease in chondrocytes^16,17^. EVs from horse amniotic mesenchymal-derived cells contain miRNAs that have potential effects, for instance, on interleukin signaling and inflammatory processes^18^. Data on the effects of OA on HA concentrations are variable in horses with unchanged or reduced HA levels and stabile or impaired viscoelastic properties of OA SF^19,20^. HA treatments can have beneficial influence on the health of joint tissues, including chondroprotection and anti-inflammatory effects^21,22^.

Alterations in the levels of SF HA and EVs may contribute to the pathogenesis of OA and have potential as joint disease biomarkers and therapeutic targets^5,23^. Based on this, the aim of the present study was to characterize SF EVs and their HA cargo in the naturally occurring equine OA model. It was hypothesized that (*i*) SF EV counts would be elevated in OA, (*ii*) HA concentrations and molecular weight would reduce in OA SF, and (*iii*) HA–EV counts would decrease in OA SF compared to control joints.

## Methods

### Animals and sampling

**Figure 1:**
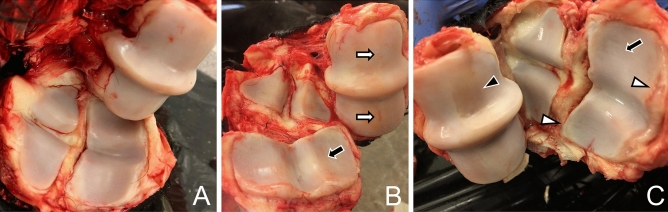
Representative photographs of articular cartilage in normal and osteoarthritic horse fetlock joints. A = normal, B = mild osteoarthritis (OA), C = severe OA, black arrows indicate thinning and wear lines in articular cartilage, white arrows indicate erosion, black arrowhead points to missing cartilage, and white arrowheads indicate osteophytosis.

Approximately 3–10 ml of SF was aspirated from arthritic metacarpophalangeal joints of 11 horses (Table 1) at the Veterinary Teaching Hospital, University of Helsinki, after medically-induced euthanasia because of OA or due to other diagnoses (spinal or dental pathology, back pain, puncture wound in hoof, leg injury, guttural pouch mycosis). The protocols included 0.1–0.2 mg/kg detomidine hydrochloride, 0.1–0.2 mg/kg butorphanol tartrate, 0.05–0.1 mg/kg midazolam, 2.2 mg/kg ketamine hydrochloride, and T-61 euthanasia solution. The OA diagnosis was based on *post-mortem* macroscopic findings on the joint surfaces (presence of wear lines, erosion of articular cartilage, osteophytes)^24^ photographed and assessed by experienced equine veterinarians (Fig. 1). Contralateral (CL) fetlock joints were also sampled, and control samples were acquired from 7 age-matched horses and one pony (Table 1) euthanized for diagnoses other than OA (colic, sinusitis, wound, neurological disease, leg injury). In the *post-mortem* examination, the fetlock joint surfaces of control animals either had no macroscopic abnormalities or revealed mild periarticular changes.

**Table 1:** Breeds and gender of the sampled horses.

	Control	CL	OA
Standardbred, n		1	1
Warmblood, n	5	9	9
Finnhorse, n	2		1
Pony, n	1		
Mare, n	5	5	6
Gelding, n	3	5	5
Horses, n	8	10	11
Joints, n	10	10	11

CL = contralateral, OA = osteoarthritis.

The average age of the horses was 11 ± 1 years (range 4–20 years) and the body mass (BM) 518 ± 13 kg (400–600 kg). Information about the medication could not be retrieved for every individual, and 3 control and 3 CL/OA horses had been treated with nonsteroidal anti-inflammatory drugs (NSAIDs). In addition, 1 control and 2 CL/OA horses had received antibiotics and 1 CL/OA horse had been treated with antifungals. The joints were classified into diagnosis groups including control (n = 7–8 depending on analysis), CL (n = 9–10), and OA (n = 9–10) based on the *post-mortem* examination of the joint surfaces. As most CL joints were noted to have mild OA, the joints were also scored according to OA severity as follows: 0 = normal (n = 6–8), 1 = mild OA (n = 8), 2 = moderate OA (n = 4–5), and 3 = severe OA (n = 7–8). The SF samples were immediately frozen in liquid nitrogen and stored at − 80 °C until analyzed.

### Nanoparticle tracking analysis (NTA)

For NTA, SF samples (1 ml, n = 26) were processed as follows: centrifugation at 1000 × *g* for 10 min, centrifugation of the supernatant at 2000 × *g* for 20 min, ultracentrifugation of the supernatant at 110,000 × *g* for 2 h and again for 90 min to wash the pellet, followed by its resuspension in 100 µl of 0.1 µm-filtered phosphate buffered saline (PBS). The Optima LE-80 K ultracentrifuge with the Ti 50.2 rotor and k-factor of 143.3 (Beckman Coulter, Brea, CA, USA) was used for ultracentrifugation.

These enriched EV samples were analyzed by NTA using the NanoSight model LM14 (Malvern Panalytical, Malvern, UK) equipped with blue laser (404 nm, 70 mW) and the sCMOS camera (Hamamatsu Photonics, Hamamatsu, Japan). The samples were diluted in 0.1 µm-filtered PBS to obtain 40–100 particles/view, and five 30-s videos were recorded using camera level 14. The data were analyzed using the NTA software *v*3.0 with the detection threshold 5 to track as many particles as possible with minimal background. After the first analysis, the samples were treated with sodium dodecyl sulphate (SDS) to lyse the EVs^25^ and re-run allowing the estimation of the proportion of EVs in the total particle counts^13^.

### Confocal laser scanning microscopy (CLSM)

The visualization of SF EVs with CLSM was performed as described previously^26^. In brief, the plasma membranes of EVs were stained with CellMask Deep Red plasma membrane stain (Thermo Fisher Scientific, Waltham, MA, USA) that was soluted together with Alexa Fluor 568-labeled HA binding complex (HABC) to visualize HA^27^. Phalloidin-iFluor, conjugated to Alexa Fluor 594 (Abcam, Cambridge, UK), was used to validate the visualized structures as actual EVs. CellMask and HABC stainings were first performed on unprocessed SFs (n = 29), and then on the same samples centrifuged at 1000 × *g* for 10 min to deplete any cell fragments. For phalloidin staining, SF was (ultra)centrifuged (1000 × *g* for 10 min, 1200 × *g* for 20 min, < 110,000 × *g* for 90 min), and the ibidi coverglass the EV fraction was placed on was coated with poly-D-lysine hydrobromide. The Zeiss Axio Observer inverted microscope equipped with the Zeiss LSM 800 confocal module was used for imaging, and the initial image acquisition was carried out using the ZEN 2.3 blue edition software (Carl Zeiss MicroImaging GmbH, Jena, Germany). The area and intensity of the stainings, particle counts, and their size distribution were determined with the ImageJ/Fiji software (NIH, Bethesda, MA) as outlined previously^26^.

### HA determinations

The total HA concentration in SFs (n = 26) was determined with a sandwich-type enzyme-linked sorbent assay (ELSA) and the HA molecular weight distribution with size-exclusion chromatography (SEC), as described previously with slight modifications^26^.

### Statistical analyses

The power analysis was conducted using the IBM SPSS *v*27.0 program (IBM, Armonk, NY, USA) with the Bonferroni correction for the estimation of statistical power separately for the general variables, NTA, CLSM, and HA data. The obtained power values varied between 0.954 and 1.000, all well above the conventional limit for reliable testing to avoid type II errors, 0.8. Comparisons between the study groups were performed with the generalized linear model (GLM), and the sex ratios were tested with the Fisher’s exact test (IBM SPSS *v*25.0 software). Correlations with OA grading were calculated with the Spearman correlation coefficient (r_s_). The *p* value < 0.05 was considered statistically significant. The results were presented as the mean ± SE. The discriminant analysis was performed separately for the different data types (NTA, CLSM, and HA data) to assess how clearly the samples in the study groups differed from one another, which variables separated them most clearly, and how well the analysis was able to classify the samples into their respective diagnoses.

### Ethics approval

Informed owner consent was obtained for each horse, and ethical approval was provided by the Viikki Campus Research Ethics Committee of the University of Helsinki (Statement 1/2018).

## Results

### General variables

There were no statistically significant differences between the ages or sex distribution according to diagnosis group or OA grade (GLM, Fisherʼs exact test, *p* > 0.05). The average BMs were higher in CL/OA horses compared to controls (544 ± 12 *vs.* 492 ± 22 kg; GLM, *p* = 0.023), and this was also observed for grade 1 OA (541 ± 12 kg) and grade 3 OA (544 ± 14 kg) *vs.* grade 0 OA (481 ± 25 kg; *p* = 0.017). The CL joints showed significantly lower scores in OA grading (1.2 ± 0.20) than the OA joints (2.8 ± 0.13; GLM, *p* < 0.001).

### Count and size distribution of small EVs in horse SF

**Figure 2:**
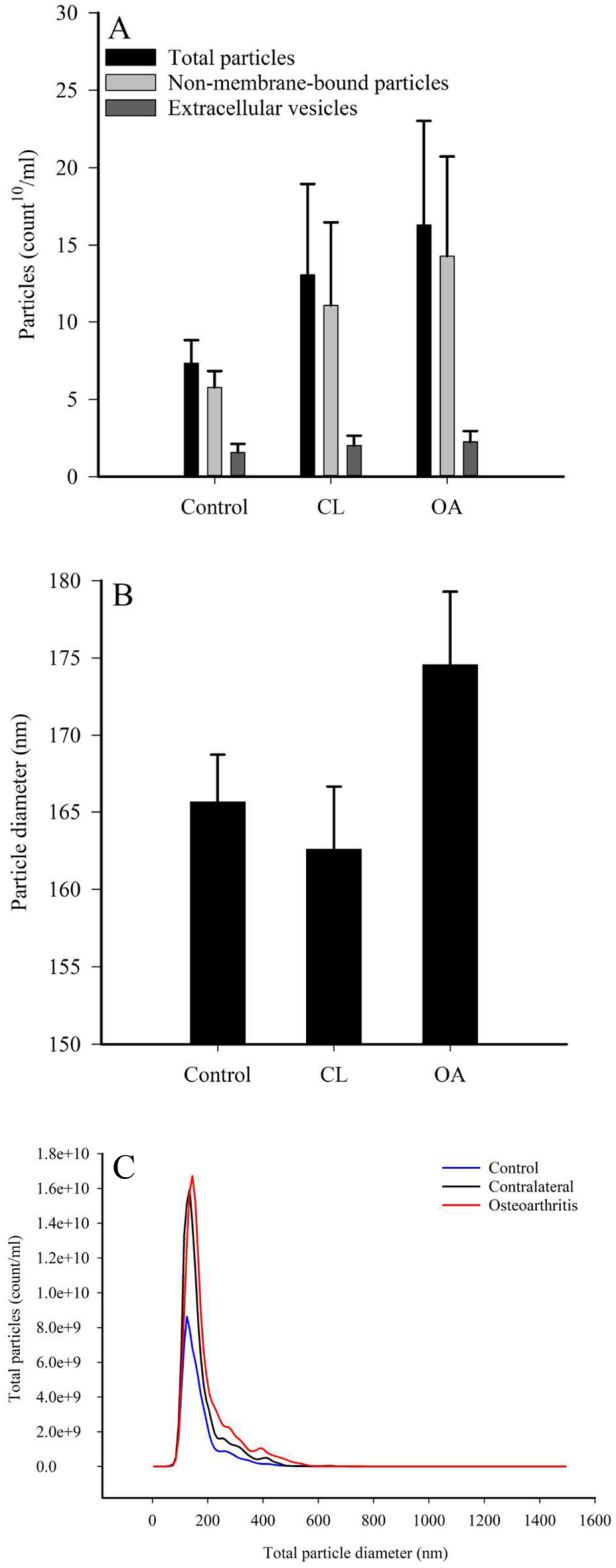
Particle counts (**A**), diameters of total particles (**B**), and size distribution of total particles (**C**) in equine synovial fluids. The samples were collected from control, contralateral (CL), and osteoarthritic (OA) fetlock joints and measured by nanoparticle tracking analysis (means + SE or means). There were no statistically significant differences between the groups (generalized linear model, *p* > 0.05).

The total particle count averaged 12.0 × 10^10^ ± 3.28 × 10^10^ particles/ml in horse SF by NTA (Fig. 2A). After the detergent treatment, it was observed that most of these particles were non-vesicular material and the estimated concentration of EVs was 1.8 × 10^10^ ± 0.32 × 10^10^ particles/ml. The majority of the particles were 100–200 nm in diameter, and there were no statistically significant differences in their count, diameter, or size distribution according to diagnosis or OA severity (GLM, *p* > 0.05; Fig. 2A–C). The discriminant analysis classified 87.5% of the samples correctly into their respective diagnosis groups based on the NTA data, and the discriminant functions 1–2 explained 100% of the variation in the dataset.

### Count and size distribution of large EVs in horse SF

**Figure 3:**
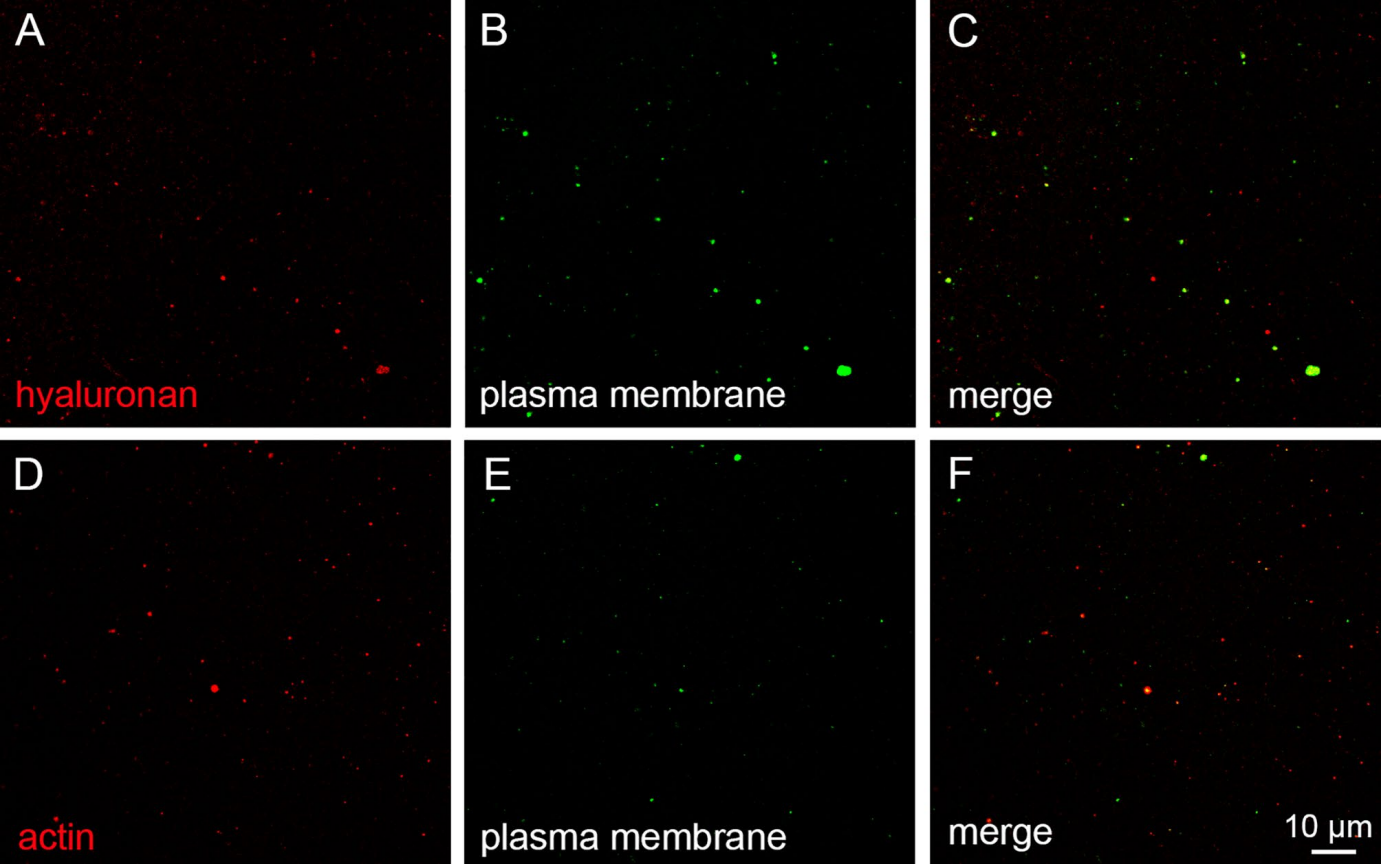
Equine synovial fluid (SF) stained with CellMask Deep Red plasma membrane stain, Alexa Fluor 568-labeled hyaluronan binding complex (HABC), and Alexa Fluor 594-labeled phalloidin. An unprocessed SF sample stained with HABC (pseudocolored red) and CellMask (pseudocolored green) in panels (**A** and **B**), an ultracentrifuged SF sample stained with phalloidin (pseudocolored red) and CellMask (pseudocolored green) in panels (**D** and **E**), and merged images in panels (**C** and **F**) depicting the co-localization of the stains.

CLSM verified that the horse SF samples contained spherical EVs and that HA and phalloidin (a marker of filamentous actin) were co-localized with the plasma membrane stain (Fig. 3). With CLSM, we documented an EV population, the diameter of which was mostly < 1000–2000 nm, while the visualized HA-particles averaged < 1000 nm in diameter. The size distributions of both EVs and HA-particles were generally similar in all diagnosis groups (GLM, *p* > 0.05). Centrifugation of SF at 1000 × *g* for 10 min caused decreases in the area, diameter, and intensity of membrane-fluorescent material (*i.e.*, EVs), co-localization of EV and HA fluorescences, and HA–EV count (*p* = 0.011–0.041).

**Figure 4:**
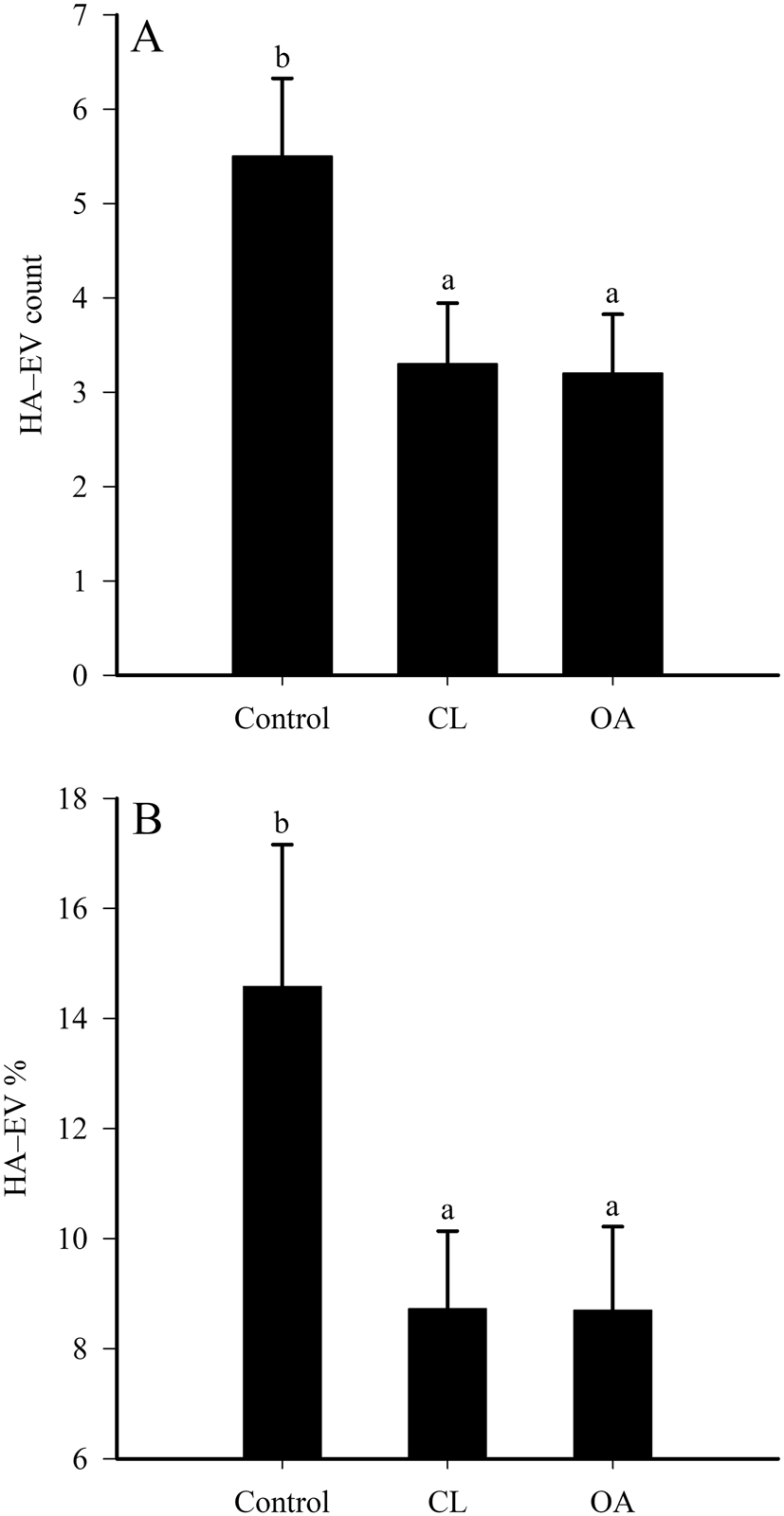
Count (**A**) and % (**B**) of hyaluronan-containing extracellular vesicles (HA–EVs) in equine synovial fluids. The samples were collected from control, contralateral (CL), and osteoarthritic (OA) fetlock joints (means + SE). HA–EV % represents the proportion of EVs with HA staining of the total EV count on the visual fields. Bars with dissimilar lowercase letters differ significantly from each other (generalized linear model, *p* < 0.05).

In respect to diagnosis group, EV diameter was higher in OA SF (1575 ± 56 nm) compared to control SF (1402 ± 34 nm; GLM, *p* = 0.034). Approximately 10.4 ± 1.09% of EV fluorescence was co-localized with HA fluorescence. HA–EV count and % were lower in CL and OA SFs than in control SF (*p* = 0.024–0.028; Fig. 4A–B). HA–EV count also decreased in grade 3 OA compared to grade 0 samples (*p* = 0.012). There were no differences in EV or HA counts, areas of EVs or HA-particles, intensities of EV or HA fluorescences, HA-particle diameter, and area or diameter of HA–EVs (*p* > 0.05). There was an inverse correlation between OA grade and EV count, HA–EV count, and HA–EV % (r_s_ = –0.264 to –0.327, *p* = 0.012–0.045). The association was positive for EV area and EV diameter with OA grade (r_s_ = 0.272, *p* = 0.039 for both).

**Figure 5:**
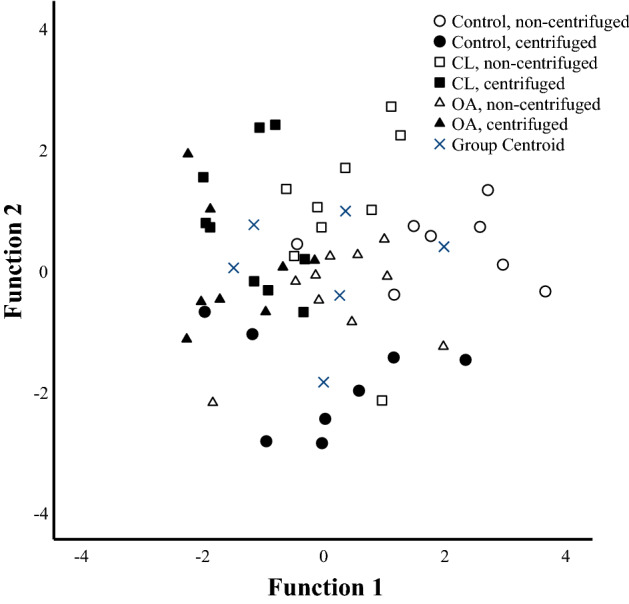
Discriminant analysis depicting the classification of confocal laser scanning microscopy data in equine synovial fluids. The samples were collected from control, contralateral (CL), and osteoarthritic (OA) fetlock joints, and the data classified based on discriminant functions 1 (on x-axis) and 2 (y-axis) that together explain 57.5% of the variance in the dataset. The samples were analyzed unprocessed and centrifuged at 1000 × *g* for 10 min prior to re-run.

For the discriminant analysis, the SF samples were divided into 6 subgroups (3 diagnoses with or without centrifugation) but neither diagnosis nor processing led to a separation of the samples into discrete clusters (Fig. 5). The variables of interest contributing to the model included HA–EV count and HA–EV %. The functions 1–5 explained 100% of the variance in the dataset, and the analysis classified 71.2% of the samples correctly into their respective diagnosis groups.

### HA concentrations in SF

**Figure 6:**
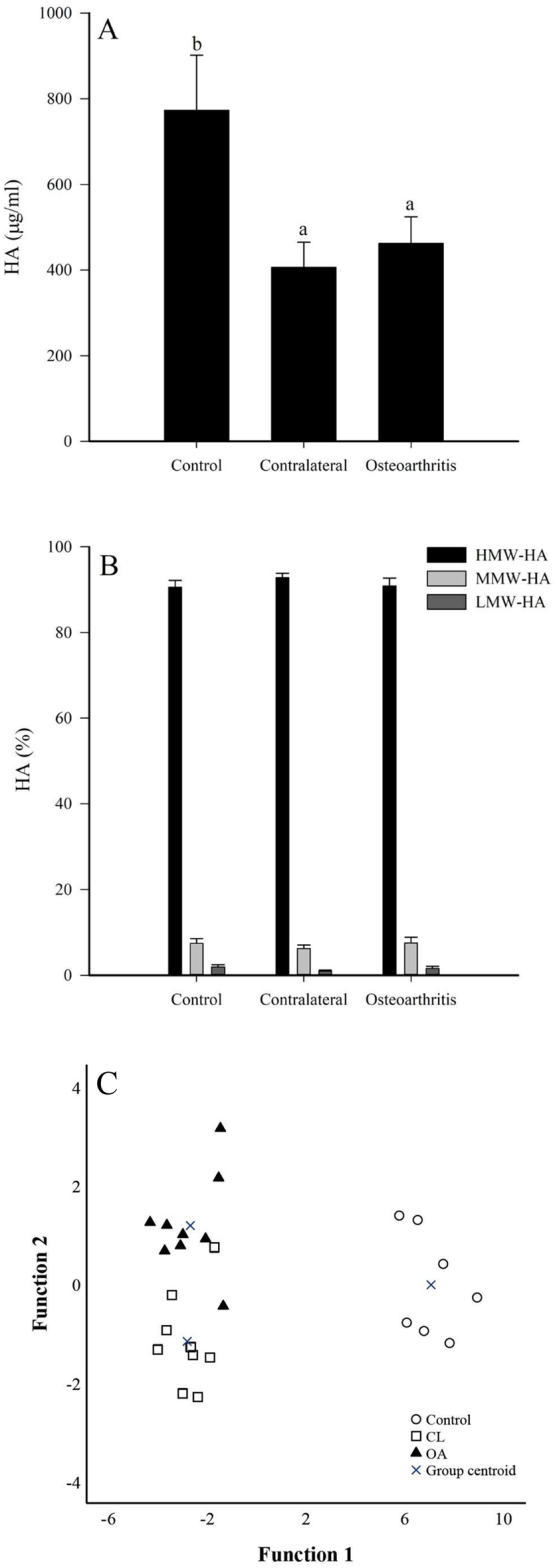
Total hyaluronan (HA) concentrations (**A**) and size distribution (**B**) in equine synovial fluids from control, contralateral (CL), and osteoarthritic (OA) fetlock joints (means + SE). Bars with dissimilar lowercase letters differ significantly from each other (generalized linear model, *p* < 0.05). Discriminant analysis (**C**) depicting the classification of HA data in equine synovial fluids based on discriminant functions 1 (on x-axis) and 2 (y-axis) that together explain 100% of the variance in the dataset. HMW = high-molecular-weight (≈2500 kDa), MMW = medium-molecular-weight (≈500 kDa), LMW = low-molecular-weight (≈150 kDa).

The total HA concentrations were lower in CL and OA joints compared to controls (GLM, *p* = 0.002; Fig. 6A) and in OA grades 1–3 compared to grade 0 (*p* = 0.021). The size distribution of HA fractions with different molecular weights was mostly similar in all diagnosis groups and, on average, > 90% of HA was of high molecular weight (≈2500 kDa; Fig. 6B).

In the discriminant analysis, control joints were clearly separated from CL and OA joints that partly clustered together based on the HA data (Fig. 6C). The total HA concentration (μg/ml) was more pivotal for the separation of the groups than the fractions with different molecular weights. The first function explained 95.1% of the variance in the dataset and the second function accounted for 4.9% of the variance. The analysis classified 92.0% of the samples correctly into their respective diagnosis groups.

## Discussion

In the present study, we characterized SF EV populations and determined HA levels in the naturally occurring equine OA model by various methods (NTA, CLSM, ELSA, and SEC). CLSM verified that the SF samples contained spherical membrane-coated particles with filamentous actin, a cytosolic protein recovered in EVs^28^. The main findings were as follows: (*i*) the number and size distribution of small EVs measured by NTA were unaffected by OA, (*ii*) larger EVs determined by CLSM increased in size but tended to decrease in number due to OA, (*iii*) the number and proportion of HA–EVs decreased in OA SF, (*iv*) total HA concentrations reduced in OA SF, and (*v*) CL joints with milder OA resembled OA joints in their EV and HA data.

Leukocytes and FLSs are among cellular sources of SF EVs, the quantity and composition of which can be altered in human patients with OA and rheumatoid arthritis (RA)^5,29^. In the present study using equine SF, the average counts and diameters of total particles detected by NTA were not affected by OA. Generally speaking, simply using NTA results as diagnostic or prognostic tools would be inadequate. This is due to methodological limitations, as NTA cannot distinguish EVs from non-membrane-bound particles^30^, which would cause a confounding factor in the analysis. In fact, the treatment of the ultracentrifuged fraction with SDS demonstrated that most of the detected particles did not represent vesicular structures. The diameter of membrane-bound particles was mostly > 100 nm in our preparation suggesting that this EV population mainly consisted of MVs. Previous studies have reported increases in cytokine- and chemokine-rich EXOs from early to severe knee OA^31^ or no differences in EV concentrations between the SFs of OA and non-OA human patients^32^. Research on EVs in equine OA SF is still in its early stages, but their counts, especially those with CD44, increased in experimentally-induced synovitis^11^. This was different from the unaffected counts of small EVs in our naturally occurring OA model, emphasizing the need for both model types in horse EV research. It would be possible to optimize the EV recovery from equine SF by pre-treatment of samples with hyaluronidase^11^, the lack of which may have reduced the counts of especially CD44^+^ EVs in the present study.

A large proportion of previous studies has focused on EXOs, but biofluids also contain other subpopulations of EVs that presumably have different characteristics and functional properties. According to Jimenez et al.^33^, small EVs (EXOs) could promote cell adhesion, while larger EVs (MVs) were enriched in proteins associated with ribosomes, RNA biogenesis and processing, and metabolism. EVs with a diameter > 1000 nm have traditionally been considered ABs, but it is now known that they can also be produced by viable cells^34^. The data on the cargo and function of these large EVs are scarce, as they are often routinely discarded by EV isolation techniques. However, human RA SF was documented to contain two subpopulations of EVs, small (100–300 nm) and large (700–3000 nm), the latter of which was associated with immune complexes^35^. In the present study, we also observed a subpopulation of large EVs in equine SF by CLSM, and OA was noted to cause a slight increase in the average diameter of these EVs. There was also an inverse association between the EV number and OA grade. A change in particle size is not routinely observed due to OA^31,32^, but Foers et al.^29^ documented lower EV concentrations but higher diameters in OA SF compared to RA SF with high-level inflammation. In human patients with autoimmune systemic lupus erythematosus, plasma contained elevated levels of large EVs (700–3000 nm)^36^ and, regarding horse plasma, endurance racing tended to increase the EV count and diameter^37^. The reason for the observed slight increase in the EV size remains unknown but it could be speculated to be related to, for instance, elevated proportions of ABs in conditions with high inflammation or physical exercise.

The present study displayed reduced HA–EV counts and % in SF due to equine OA, which was not previously documented for human SF with the same CLSM technique^26^. Different cell types can release HA–EVs but their functions remain unknown^38^. They have been hypothesized to carry HA to distant sites, be able to synthesize new HA, transport signals to target cells, function in tissue remodeling and regeneration, and affect motility and metastasis of cancer cells. By transporting bioactive cargo, HA–EVs may also participate in inflammatory or pro-resolving processes in inflamed joints. In the present sample material, the proportion of HA–EVs of total EVs was approximately 12% in unprocessed SF and lower than measured previously in humans^26^. If the reduction in the HA–EV counts could be shown to be a general feature of equine OA SF, HA–EVs may have potential as a joint disease biomarker and therapeutic target, as their levels can be monitored with CLSM by using simple stainings. The analysis does not require a great deal of post-sampling processing, but the access to specialized equipment would limit the applicability of HA–EVs as a biomarker. For translational studies, the factors modifying EV secretion in inflammation could be examined in the future and/or EV preparations could be given as therapeutic agents. It remains to be investigated if the reduction of HA cargo could justify the supplementation of HA in equine OA joints. Despite well-established beneficial effects, there is emerging controversy regarding the clinical effectiveness of intra-articular HA administrations^39^. In fact, recent integration of meta-analysis with bioinformatics revealed that HA could also induce local and systemic inflammatory responses and may not be ultimately beneficial as an OA treatment.

Previously, when equine chondrocytes were incubated with SF EVs, genes involved in joint inflammation were downregulated, thus, implying anti-OA effects^11^, and indications of decreased cartilage degradation by bone marrow MSC-derived EVs were documented in other recent horse studies^16,17^. The present experiment observed a loss of HA–EVs in equine OA SF that is known to be of inflammatory nature^8^, but it remains to be established if HA–EVs would be beneficial for the joint health or if they could play a role in disease pathogenesis. EVs derived from inflammatory cells and chondrocytes can activate anabolic gene expression and reduce the expression of cartilage-degrading proteinases in chondrocytes, resulting in the deposition of extracellular matrix and cartilage protection^40,41^. They can also polarize macrophage response towards an anti-inflammatory phenotype in cartilage and synovium and attenuate the development of experimentally-induced arthritis in animal models. Especially MSC-derived EVs can alleviate inflammation and pain as well as induce cartilage repair^5^. The potential roles HA–EVs could play in inflammation/resolution and degradation/repair in the joint environment should be investigated in future studies. Previous research has shown that intra-articular injections of high-molecular-weight HA combined with stem cell-derived EVs can improve cartilage repair and regeneration compared to HA alone^42^. The observations of the changes in HA–EVs in joint disease add to this knowledge and offer a new direction of EV research in OA of human and animal patients.

It was observed in the present study that the centrifugation of horse SF at 1000 × *g* for 10 min decreased the area, diameter, and intensity of EV fluorescence, co-localization of EV and HA fluorescences, and HA–EV count. While centrifugation is useful in removing larger particles, such as cellular debris, it also changes the characteristics of the desired research targets. Thus, it is of importance to examine not only processed samples but also biological fluids in their original state to gain comprehensive knowledge on EV biology. It was previously reported that the cellular origin of EVs can determine how they are affected by centrifugation and, for instance, the centrifugation of human plasma at 18,890 × *g* for 2 h at 20 °C resulted in the aggregation of EVs^43^. Our findings suggest that even low-speed centrifugation could influence the characteristics and cargo of EVs, which highlights the need for careful reporting of all steps of EV sample processing. Furthermore, it is becoming clear that unprocessed, centrifuged, and ultracentrifuged samples reflect different particle populations in SF, and while different centrifugation protocols can assist in the visualization of smaller particles, the populations of larger EVs are either decreased or lost and, thus, their potential biological significance would remain uninvestigated. We tentatively suggest that it would be prudent to continue assessing various EV populations at this stage of equine EV research.

Regarding the conventional measurement of HA concentrations, the total HA levels decreased in both OA and CL SFs, while the molecular size distribution of HA remained unaffected. These results agree with previous data from OA horses with unchanged or decreased HA levels and stabile or impaired viscoelastic properties of SF^19,20^. HA is utilized in OA therapies of both human and equine patients, as it can induce chondroprotection and anti-inflammatory effects^14^. SF HA may also influence the uptake of EVs into joint tissues and, thus, mediate cell-to-cell communication. EVs have been shown to enter, for instance, synoviocytes, and the HA matrix around FLSs facilitates EV internalization, which may be partly mediated via CD44 on the EV surface^44^. In fact, CD44^+^ EVs became elevated in the SF of horses with experimentally-induced synovitis^11^.

The present study documented similarities in the EV and HA data between the CL and OA joints of horses. In studies using experimentally-induced OA models, CL knees are sometimes used as within-animal controls^45^, but it is known that their biomechanical properties can be altered^46^ and, in a rabbit model, several types of differences were documented in joint tissues between control and CL knees^47–49^. For this reason, we expected that the characteristics of CL SF would differ from control joints, and the *post-mortem* examination of joint surfaces confirmed that mild OA was present in most of the CL joints^23^. This emphasizes the need for a thorough examination of the CL joint, as it cannot be considered unaffected by the OA joint or be uncritically used for comparison as a control. It is well established in human studies that unilateral knee OA is a risk factor for developing bilateral knee OA^50^. The grading scores of the CL joints were clearly lower than those of the OA joints, but they still exhibited the reduced HA–EV counts. This could provide future diagnostics and prognostics a useful tool to detect early-stage OA and to implement appropriate measures (such as level of exercise) to prevent further damage on the joint.

There are some limitations to be considered in the present study. The number of animals was relatively small, as it was difficult to obtain euthanized horses with unaffected fetlock joints. For this reason, one Estonian riding pony was included in the control group. Despite some variation in the assignment of the different breeds into the experimental groups, there was no statistical difference in their distribution. It was also impossible to determine if the diagnosed OA cases were spontaneous, of post-traumatic origin or, in one case, initiated by osteochondrosis. A few animals were euthanized for OA, while the remaining OA horses had only mild symptoms and OA was more of an incidental finding in these cases. Intra-articular medication was not administered to the animals, but 6 horses had been treated with NSAIDs, which may have influenced EV populations, albeit literature about the possible interactions between NSAID use and EV release remains scarce^51^. Finally, the SF samples were not pre-treated with hyaluronidase before ultracentrifugation, which could have reduced the recovery of especially CD44^+^ EVs for NTA^11^.

## Conclusion

The diagnosis of early OA still lacks specific molecular markers in body fluids for both humans and horses. The present study discovered a potential biomarker candidate for naturally occurring equine OA. The observed reduction in large HA–EVs could provide an additional tool for the diagnosis of early-stage OA before this debilitating condition becomes symptomatic or radiological signs develop. Eventually this would allow the development of early-stage measures to counteract the cartilage destruction that is the hallmark of inflammatory joint diseases. Based on the results of the present study, the association of HA–EVs with other related diseases also merits further research.

## Data Availability

All relevant data generated or analyzed during this study are included in this published article.

## Abbreviations

AB: Apoptotic body
BM: Body mass
CD44: Cluster of differentiation 44
CL: Contralateral
CLSM: Confocal laser scanning microscopy
ELSA: Enzyme-linked sorbent assay
EV: Extracellular vesicle
EXO: Exosome
FLS: Fibroblast-like synoviocyte
GLM: Generalized linear model
HA: Hyaluronan
HABC: Hyaluronan binding complex
HA–EV: Hyaluronan-containing extracellular vesicle
MV: Microvesicle
MSC: Mesenchymal stem cell
NSAID: Nonsteroidal anti-inflammatory drug
NTA: Nanoparticle tracking analysis
OA: Osteoarthritis
PBS: Phosphate buffered saline
RA: Rheumatoid arthritis
r_s_: Spearman correlation coefficient
SDS: Sodium dodecyl sulphate
SEC: Size-exclusion chromatography
SF: Synovial fluid
